# Supplementary material for: Use of social media platforms by migrant and ethnic minority populations during the COVID-19 pandemic: a systematic review
Source: BMJ Open. 2022 Nov 16;12(11):e061896. doi: 10.1136/bmjopen-2022-061896 (PMC9676419; doi:10.1136/bmjopen-2022-061896)
Supplement: Supplementary data [file bmjopen-2022-061896supp001.pdf]

Supplementary file 1

Online supplement 1: Search Strategy

We accessed the WHO COVID-19 Database using the following link:

<https://search.bvsalud.org/global-literature-on-novel-coronavirus-2019-ncov/>

We used the advanced search available at:

< https://search.bvsalud.org/global-literature-on-novel-coronavirus-2019-ncov/advanced/?lang=en >

We entered the following search terms into separate boxes linked by the 'AND' term. We specified that the following terms must appear in the title, abstract or subject.

Migrant and ethnic minorities	Ancest* OR Diaspor* OR ethnic* OR Ethnoc* OR Ethnog* OR "Identity politics" OR Ingroups OR outgroups OR Intersectionality OR Kinship OR "Minority group*"~3 OR "minority population*"~2 OR minorities OR Multicultu* OR Polyethnic* OR "Population genetics" OR Race OR races OR racial OR Tribe* OR latino*) OR AB:(Ancest* OR Diaspor* OR ethnic* OR Ethnoc* OR Ethnog* OR "Identity politics" OR Ingroups OR Outgroups OR Intersectionality OR Kinship OR "Minority group*"~3 OR "minority population*"~2 OR minorities OR Multicultu* OR Polyethnic* OR "Population genetics" OR Race OR races OR racial OR Tribe* OR latino*) OR "afro american*"~3 OR BAME OR latino* OR roma OR romani OR refugee* OR immigrant* OR "migrant" OR "displaced person" OR "displaced persons" OR "social determinant*"~2 OR "latin population" OR "latin group*" OR "people of color" OR "people of colour"
AND	AND
Social media	social media OR social network OR online communit* OR online discuss* OR online communicat* OR online post OR messag* OR chat OR media OR misinformat* OR disinformat* OR malinformat* OR fake new* OR twitter OR whatsapp OR tweet OR post OR instagram OR reddit OR weibo OR sina OR youtube OR tiktok OR snapchat OR pinterest OR likee OR sharechat OR

	discord OR kuaishou OR wechat OR weixin OR qq OR telegram OR quora OR mobile app OR blog OR podcast OR hashtag OR antivax* OR vaccine hesitanc* OR web 2.0 OR online

No additional filters or limits were used.

The WHO COVID-19 Database gathers the latest international multilingual scientific findings and knowledge on COVID-19. The global literature cited in the WHO COVID-19 database is updated daily (Monday through Friday) from searches of bibliographic databases, hand searching, and the addition of other expert-referred scientific articles. This database represents a comprehensive multilingual source of current literature on the topic.

The WHO COVID-19 Database draws literature from the following databases: Embase, Web of Science, Oxford Academic Journals, PubMed NIH, Clinical Trials, China CDC MMWR, CDC reports, ProQuest Central (Proquest), CINAHL, Africa Wide Information (Ebsco), Scopus, PsycInfo, CAB Abstracts, Global Health, J Stage, Science Direct, Wiley Online Journals, JAMA Network, British Medical Journal, Mary Ann Liebert, New England Journal of Medicine, Sage Publications, Taylor and Francis Online, Springer Link, Biomed Central, MDPI, ASM, PLOS, The Lancet, Cell Press, and pre-print sites chemRxiv, SSRNbioRxiv, and medRxiv.
