# Supplementary figures and images for: Use of social media platforms by migrant and ethnic minority populations during the COVID-19 pandemic: a systematic review
Source: BMJ Open. 2022 Nov 16;12(11):e061896. doi: 10.1136/bmjopen-2022-061896 (PMC9676419; doi:10.1136/bmjopen-2022-061896)

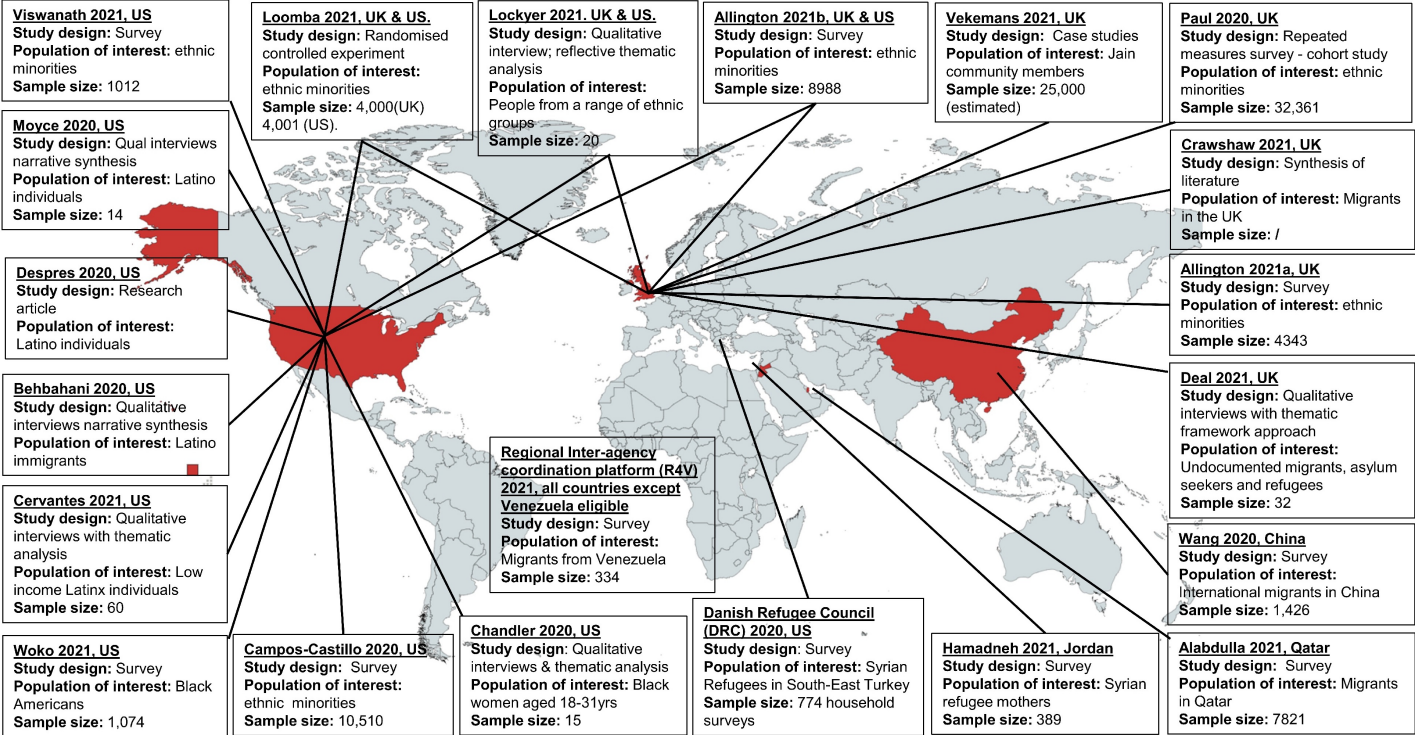

Supplement: Supplementary data [file bmjopen-2022-061896supp002.pdf]
